# Hidden neural networks for transmembrane protein topology prediction

**DOI:** 10.1016/j.csbj.2021.11.006

**Published:** 2021-11-08

**Authors:** Ioannis A. Tamposis, Dimitra Sarantopoulou, Margarita C. Theodoropoulou, Evangelia A. Stasi, Panagiota I. Kontou, Konstantinos D. Tsirigos, Pantelis G. Bagos

**Affiliations:** a Department of Computer Science and Biomedical Informatics, University of Thessaly, 35100 Lamia, Greece; b Institute for Translational Medicine and Therapeutics, University of Pennsylvania, Philadelphia, Pennsylvania, USA; c Present address: National Institute on Aging, National Institutes of Health, Baltimore, Maryland, USA.; d EMBL-EBI, Wellcome Genome Campus, Cambridge, United Kingdom

**Keywords:** HMM, Hidden Markov Models, HNN, Hidden Neural Networks, NN, Neural Networks, CHMM, Class Hidden Markov Models, ML, Maximum Likelihood, CML, Conditional Maximum Likelihood, JUCHMME, Java Utility for Class Hidden Markov Models and Extensions, EM, Expectation-Maximization, MSA, Multiple Sequence Alignment, SOV, segment overlap, MCC, Matthews Correlation Coefficient, Hidden Markov Models, Hidden Neural Networks, Membrane proteins, Sequence analysis, Neural Networks, Protein structure prediction

## Abstract

Hidden Markov Models (HMMs) are amongst the most successful methods for predicting protein features in biological sequence analysis. However, there are biological problems where the Markovian assumption is not sufficient since the sequence context can provide useful information for prediction purposes. Several extensions of HMMs have appeared in the literature in order to overcome their limitations. We apply here a hybrid method that combines HMMs and Neural Networks (NNs), termed Hidden Neural Networks (HNNs), for biological sequence analysis in a straightforward manner. In this framework, the traditional HMM probability parameters are replaced by NN outputs. As a case study, we focus on the topology prediction of for alpha-helical and beta-barrel membrane proteins. The HNNs show performance gains compared to standard HMMs and the respective predictors outperform the top-scoring methods in the field. The implementation of HNNs can be found in the package JUCHMME, downloadable from http://www.compgen.org/tools/juchmme, https://github.com/pbagos/juchmme. The updated PRED-TMBB2 and HMM-TM prediction servers can be accessed at www.compgen.org.

## Highlights

• A method that combines HMMs and Neural Networks for biological sequence analysis.
• The updated versions of PRED-TMBB2 and HMM-TM outperform the currently available methods.
• The JUCHMME library – an open-source CHMM library contains the HNN implementation.
• The JUCHMME is the only publicly available implementation of HNNs.

## Graphical abstract

**Figure:**
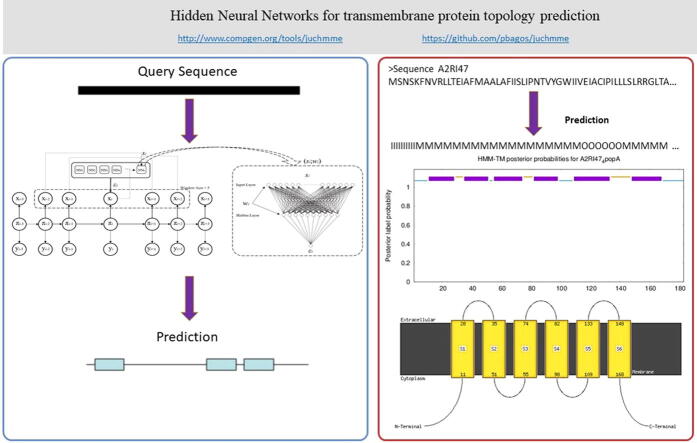

## 1 Introduction

Hidden Markov models (HMMs) are statistical models, which have been successfully applied to various problems in biological sequence analysis over the years [1]. A sequence being modeled by a standard HMM assumes a Markov process with unobserved hidden states which, in its basic formulation, operates in an *unsupervised* manner. However, many applications in molecular biology, which include protein structure prediction and gene-finding, need a *supervised* learning procedure such as Class Hidden Markov Models (CHMM) [2]. In this case, a sequence of labels (**y**) accompanies each observation sequence (**x**), corresponding to the different attributes that we want to predict.

In general, the 1^st^ order Markovian assumption with regard to transition probabilities may not be sufficient in all cases, since the sequence surrounding a residue (the context) can potentially contain information that can augment the prediction performance. To address this issue, many extensions have been proposed. These include the higher-order HMMs (HOHMM) where a higher order (*t*^th^) Markov chain is used for the state transition probability [3], the partial HMMs (PHMM), where both transition and emission probabilities are conditioned on previous observations [4] or similar models which consider the *n* previous symbols of observations, allowing thus a context dependence among residues [5], [6]. In our previous work, we demonstrated that, by simply altering the emission probabilities, we can significantly improve the performance of existing HMM-based predictors [7]. A more general model that can incorporate all the aforementioned models as special cases is the Hidden Neural Network (HNN). HNN is a hybrid model combining the CHMM framework with Neural Networks (NNs) for building a more flexible classifier. The core idea in the HNN is to replace the probability matrices of the CHMM by NN outputs that take as input the observation sequence [8].

In this work, we apply the HNN framework to the task of transmembrane protein topology prediction and compare against the available predictors. The HNN implementation is freely available through the JUCHMME library [9].

## 2 Methods

### 2.1 Hidden Markov Models

A Hidden Markov Model (HMM) is a model for representing probability distributions over sequences of observations consisting of transitions that linked a set of states forming a Markov chain [10], [11]. More formally, suppose an aminoacid sequence **x** of a protein length *L* denoted by **x** = *x*_1_, *x*_2_, …,*x_L_*, where each observation symbol is produced by a given state (*k*) according to the emission probability *e_k_*(*x_i_*). Based on HMM parameters, we can calculate the total probability of the HMM model for a sequence  **x** using the forward (or the backward) algorithm:

(1) $$P\left(\text{x}|\theta \right)=\underset{\pi }{\sum }P\left(\text{x},\pi |\theta \right)=\underset{\pi }{\sum }a_{B\pi_{1}}\overset{L}{\underset{i=1}{\prod }}e_{\pi i}\left(x_{i}\right)a_{\pi_{i}\pi_{i+1}}$$

Typically, the training phase of a HMM is performed by the Baum-Welch algorithm [10], [11], [12], which is a special case of the Expectation-Maximization (EM) algorithm for incomplete data [13]. The algorithm estimates the transitions and emissions probabilities by Maximum Likelihood (ML) from the observed transitions and emissions using Forward and Backward algorithms. Alternative, Baldi and Chauvin proposed a gradient-descent method capable of the similar task [14]. Maximization of the likelihood, in such cases, corresponds to an *unsupervised learning* procedure.

A useful approach to modeling biological protein sequences for classifying smaller substructures, in complex biological sequence analysis problems, is to use labeled sequences for training. When using this approach for training, one can incorporate a sequence of labels **y** (**y** = *y*_1_, *y*_2_, …,*y_L_*) for each amino acid in position *i* of the sequence **x**. In this case, we also need the probability *δ_k_*(*y_i_* = *c*) of a state *k* having a label *c*. In most sequence analysis problems, we can use a simple delta-function, since a particular state is not expected to match more than one label. Furthermore, Krogh in his seminal paper proposed modifications to the forward and backward algorithms in order to allow training using labeled data [2]. Thus, we can now maximize the joint probability of the sequences and the labels given the model with the summation performed over the paths *Π_y_* that agree with the labels **y**:

(2) $$P\left(\text{x}\text{,}\text{y}|\theta \right)=\underset{\pi }{\sum }P\left(\text{x},\text{y}\text{,}\pi |\theta \right)=\underset{\pi \in \Pi_{y}}{\sum }P\left(\text{x}\text{,}\pi |\theta \right)$$

Since labels are used, the particular approach corresponds to *supervised learning*. Based on labeled sequences, we can also perform Conditional Maximum Likelihood (CML) estimation where the model is trained in a discriminative manner. In this approach, the probability of the labels given the sequences is maximized, *P*(**y**|**x**,*θ*) = *P*(**x**,**y**|*θ*)/*P*(**x**|*θ*) [15]. The EM algorithm cannot be used in this setting and a gradient-descent method is more appropriate [16]. To compute the gradients, we use the negative log-likelihood, where we define:

(3) $$\ell =-\log P\left(y\left|x,\theta \right)\right)=\ell_{c}-\ell_{f}$$

(4) $$\ell_{c}=-\log P\left(x,y\left|\theta \right)\right)$$

(5) $$\ell_{f}=-\log P\left(x\left|\theta \right)\right)$$

According to this approach, the above expectations *c* and *f* correspond to the two forward–backward passes for each training sequence, once in the free-running phase (*f*) and once in the clamped phase (*c*).

### 2.2 Hidden Neural Networks

Over the past decades, Neural Networks have proven extremely useful for problems within the field of biological sequence analysis. They have been applied to many important problems, ranging from protein structure prediction to sequence classification and gene identification [17]. As biological sequence analysis is essentially a pattern recognition task, several researchers started combining elements of HMMs and neural networks expecting more powerful and flexible models for classification. A general framework of hidden neural networks was introduced by Krogh and Riis [8] and was used initially for speech recognition. In the proposed hybrid model, some CHMM probability parameters are replaced by neural networks outputs that take the observations as input. In this approach, the model is trained by gradient-descent in a procedure where the neural networks are updated by backpropagation and the errors are calculated by a modified forward–backward algorithm. However, the applications of HNNs in computational biology were limited in the prediction of the disulfide bonding state of cysteines [18] and secondary structure prediction [19]. It is important to note that, in the original HNN formulation, the HMM and NN components were trained in combination, whereas, in the above-mentioned applications, the NN and HMM have been trained separately. In this respect, the current implementation follows close the original method of Krogh and Riis.

#### 2.2.1 HNN architecture

**Fig. 1:**
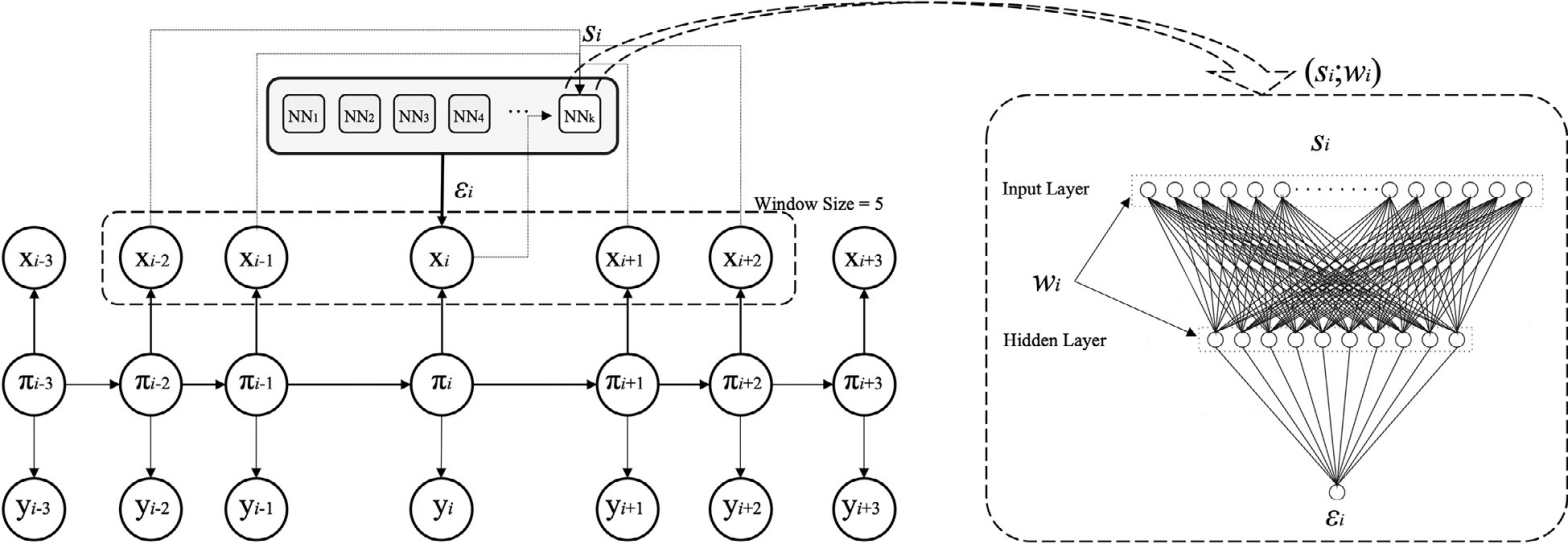
Network representations of a Hidden Neural Network (HΝΝ). In this model, the standard probability parameters are replaced by the outputs of neural networks (a symmetrical window size 5 of context around *x_i_*) assigned to each state.

The HNN used here is an instance of the original HNN method proposed by Krogh and Riis [8]. The network representation of this hybrid system is shown in Fig. 1. The basic idea in the HNN is that the standard probability parameters of a CHMM are replaced by the outputs of Neural Networks assigned to each state. For each *x_i_*, the method uses a window of context around *x_i_* corresponding to the network input *s_i_*. Defining the window size on the left and on the right, the window can be symmetrical or asymmetrical. We will denote *s_i_* the context of observation *x_i_*. The emission network in state *k* is parameterized by the weight vector *w_k_* where accepts the observation context vector *s_i_* (defined above) as input and has only one output. In cases where the context *s_i_* extends beyond the boundaries of the observation sequence, zero padding can be used to ensure a well-defined input to the networks.

In most of NNs applications in molecular biology, the architectures used are layered feed-forward architectures. In this work, the neural network in the HNN is a feed-forward multilayer perceptron network (MLP) with one hidden layer. The input layer uses the sigmoid function with a window of *K* residues. The input representations chosen to encode the sequence data could use the sparse encoding or some other alternative coding scheme. Another interesting encoding scheme is the PSSM that can be generated using any alignment program like PSI-BLAST [20] or HMMER [21]. In total, the input layer for a *K*-size window has 20 × *K* units, assuming the use of sparce encoding. For the hidden layer, we use a variable number of units. The output layer has one unit corresponding to the output probability. We can normalize the output by a standard (asymmetrical) sigmoid activation function. Furthermore, we have a large degree of freedom in the selection of hidden layer output functions where a natural choice, it appears to be a standard asymmetrical sigmoid function. Another possible choice is a sigmoid modified function or a hyperbolic tangent function, where *h* is the input to the output unit in question.

#### 2.2.2 Weight initialization

Contrary to the emission distributions in the CHMM, it is not possible to initialize the emission network weights in the HNN by the efficient Baum-Welch re-estimation algorithm. Instead of just using a set of emission networks initialized by random weights, another initialization method was tested based on interpreting the emission network outputs. A reasonable initialization is therefore performed if we train each emission network separately to classify the sequences into each of the different classes. We adjust the weights of the NN by the RPROP algorithm [22] after the training phase. RPROP is a first order optimization algorithm for supervised learning, acts independently on each weight and adjusts it whenever there is a sign change of the partial derivative of the total error function. Basically, as long as the NN is not converging, the weights change values in a higher rate with *e_d_* < 1 Eq. (6a, 7a), while the weights change slowly with *e_a_* > 1 when the sign change of the partial derivative of the total error function is small Eq. (6b, 7b). Thus, each weight adjusts the opposite way of the partial derivative of the error function that corresponds to this weight and aims at the reduction of the total error.

For every label of the CHMM we create and train a NN. The error function used for the training of the NN is Root Mean Square Error (RMSE) or Cross Entropy (CE), with CE achieving faster and better training in classification problems [23]. Then, the weights of these networks are used to initialize the HNN, which is trained with CML and Gradient Descent.

(6) $$\Delta w=\left\{\begin{array}{c} w\left(t-1\right)e_{d,}\frac{\partial E\left(t-1\right)}{\partial w}\frac{\partial E\left(t\right)}{\partial w}<0\\ w\left(t-1\right)e_{a,}\frac{\partial E\left(t-1\right)}{\partial w}\frac{\partial E\left(t\right)}{\partial w}>0 \end{array}\right)\left(a\right)\left(b\right)$$

(7) $$w\left(t\right)=\left\{\begin{array}{c} w\left(t-1\right)+\Delta_{w,}\frac{\partial E\left(t\right)}{\partial w}<0\\ w\left(t-1\right)-\Delta_{w,}\frac{\partial E\left(t\right)}{\partial w}>0 \end{array}\left(a\right)\left(b\right)\right)$$

#### 2.2.3 Training and decoding the HNN

One of the main ideas in the HNN approach is to train the model in a supervised manner, by a joint optimization of parameters. Just like the CHMM, the HNN is not possible to be trained using the EM algorithm and thus, a gradient-descent method is proposed.

Similar to Eqs. (1), (2), for the CHMM with regards to a general weight *w_k_* in the emission network assigned to state *k*, we therefore define:

(8) $$P\left(\text{x}|\theta \right)=\sum_{\pi }P\left(\text{x},\pi |\theta \right)=\sum_{\pi }a_{B\pi_{1}}\prod_{i=1}^{L}e_{\pi i}\left(s_{i};w_{\pi i}\right)a_{\pi_{i}\pi_{i+1}}$$

(9) $$P\left(\text{x, y}|\theta \right)=\sum_{\pi \in \Pi_{y}}P\left(\text{x,}\pi |\theta \right)=\sum_{\pi \in \Pi_{y}}a_{B\pi_{1}}\prod_{i=1}^{L}e_{\pi i}\left(s_{i};w_{\pi i}\right)a_{\pi_{i}\pi_{i+1}}$$

More formally, the CHMM emission probability *e_k_*(*x_i_*) of observation *x_i_* in state *k* is replaced by the output of an emission network *e_k_*(*s_i_*;*w_k_*) specific to state *k*. This emission network is parameterized by the weight vector *w_k_* where accepts the observation context vector *s_i_* as input and has only one output. The probability of the labelling is then computed by Eq. (2). Both Eq. (4) and Eq. (5) can be computed by a straightforward extension of the forward algorithm [8].

By using the forward–backward algorithm [24] we can calculate the derivative of log*P*(**y**|**x**,*θ*) resulting in a backpropagation training of the neural networks using the error signal. In this case, a forward–backward pass is needed for each sequence **x**. The gradients can be computed by using the standard backpropagation algorithm on the NNs in the HNN, where the error for each input *x_i_* is

(10) $$\varepsilon_{k}\left(x_{i}\right)=\frac{\ell_{c}-\ell_{f}}{\left(s_{i};w_{k}\right)}$$

where *s_i_*;*w_k_* is the weighted input to the output of the emission network assigned to state *k*. The total probability can be calculated using the forward, or backward, algorithm by replacing, those parameters that are estimated by neural networks. Since we need both the *f* and *c* counts, we have to run two forward–backward passes for each training sequence, once in the free-running phase (*f*) and once in the clamped phase (*c*). Furthermore, we have incorporated some standard techniques applied to the backpropagation such as weight decay and momentum [25]. In this application we used, instead of the standard gradient-descent, an algorithm presented initially for CHMMs that resembles closely the RPROP algorithm [26]. This approach allows easier and faster convergence in all cases.

Although any decoding algorithm can be used (i.e. Posterior-Viterbi, Viterbi, etc.), Viterbi decoding is not expected to perform well for discriminative methods, since the model is optimized to maximize the probability of correct labeling [27] and the Optimal Accuracy Posterior Decoder [28] seems to be the obvious choice.

#### 2.2.4 General comments

Even though the HNN is a simple extension of the standard CHMM, it is capable of building a more accurate predictor. Since the Neural Networks in the HNN can directly use the observation context as input they can exploit higher order correlations taking into consideration neighboring observation vectors. Therefore, it is possible to assign a Neural Network to each state estimating a score for how well the current observation matches the state given the observation context. The advantages of combining a HMM and a NN are that, while the NN dominates the modeling of complex functions with many parameters, the HMM is advantageous due to the precision of the first order algorithms it uses and the grammar that imposes along the sequence. Thus, the HNN, as a whole, incorporates the advantages of both techniques and manages to model the data more accurately.

A significant issue to achieve the best accuracy in the case of multi-layer networks is the choice of the optimal network size and the optimal number of hidden units. The optimal parameters are usually not known in advance and this is an area of active research, as we can see from the results taking into consideration various values of window size and network size. A potential problem in using different input contexts and different hidden units is the computational complexity. For instance, the HNN using emission networks with 7 hidden units and a symmetric window size 7 contains 20,460 parameters for the network weights. If a model has more hidden units and/or larger window size, then the network becomes more computationally expensive. However, neural networks, and multi-layer perceptrons in particular, are highly parallelisable architectures and the HNN is therefore well suited for implementation across parallelised computer architectures.

We note in passing that *s_i_* can in principle be any sort of information related to **x**. In biological sequence problems, for instance, one could imagine that other information could include hydrophobicity, charge or other physicochemical properties. Similarly, there is generally no assumption of independence between elements of continuous observation vectors. The HNN method presented here is implemented in the JUCHMME library – an open-source CHMM library based on Java [9]. JUCHMME is, to our knowledge, the only publicly available implementation of HNNs. A major advantage of JUCHMME is the ease of use and parameterization providing user full customization through a simple and well-document configuration file, without requiring programming skills.

### 2.3 Datasets and evaluation criteria

We measured the performance of our new approach on the tasks of topology prediction regarding alpha-helical membrane proteins and beta-barrel outer membrane proteins. In both cases, we also measured the ability of the predictor to discriminate from other classes of proteins. To ensure a fair comparison, each prediction method that we tested against was trained using both the standard CHMM approach and the HNN approach described here. For alpha-helical membrane proteins we used the HMM-TM predictor [29], which we re-trained on a dataset of 308 membrane proteins with known structure and transmembrane topology that were used during the development of TOPCONS2 [30]. We applied a homology reduction threshold of 30% on these proteins that resulted in 284 sequences in the set. For testing HMM-TM in the task of discriminating between alpha-helical and non-alpha-helical membrane proteins, we also used a negative dataset that contains 3597 sequences (from the TOPCONS2 method). For beta-barrels, we used the PRED-TMBB2 predictor [31] with and without homologs (MSA), trained on a non-redundant dataset of 49 beta barrel proteins with known structure and transmembrane topology (positive set) and a negative set which contains 1009 sequences (obtained from PRED-TMBB2). Since many of the proteins in our training set were also present in the sets used to train other tools, we decided to perform another benchmark using the 59 proteins used for training BetAware-Deep dataset [32]. Out of the 59 proteins, 26 were already present in PRED-TMBB2’s training set, whereas, for the remaining 33, we used the 2nd algorithm of Hobohm et al. [33] to remove sequences having more than 30% sequence similarity in a BLAST [20] alignment in a length of more than 80 residues in comparison with training sets of other tools. This procedure resulted in 7 outer membrane proteins that constitute our final blind test set.

We preserved the HMM architectures as per their original publications and used a strict 10-fold cross-validation procedure during the evaluation. Training stops when the minimum of the error on the held-back data is achieved during training. The emission networks are initialized separately by RPROP. This speeds up training of the HNN considerably and the models are less prone to get stuck in local minima. Thus, the performance on the cross-validation set reaches a maximum within less than 50 epochs for all tested models. Regarding transmembrane proteins, we evaluated the performance based on (i) the number of correctly predicted residues in a two-state mode (Q2 metric), (ii) the segment overlap (SOV), (iii) the number of correctly predicted topologies and (iv) the number of proteins with correctly predicted number of strands. Regarding the discrimination performance, we measured the specificity (i.e. how many non-TMBBs are correctly identified as such in a dataset with verified non-TMBBs) the sensitivity (i.e. the proportion of TMBBs positively identified in the datasets of known TMBBs) and the Matthews Correlation Coefficient (MCC), a metric of overall efficiency of a prediction algorithm.

### 2.4 Multiple sequence alignments

Evolutionary information that originates from Multiple Sequence Alignments (MSAs) have been widely used in bioinformatics in order to obtain a gain in prediction accuracy. In the case of PRED-TMBB2 (beta-barrels) the parameters presented in the original paper are used [31] and in the case of HMM-TM (alpha-helical membrane proteins) [29] the jackhmmer search [34] is performed against the nr50 database with an e-value cutoff of 10^−5^. We only included hits with an at least 75% coverage of the length of the query sequence.

## 3 Results

**Fig. 2:**
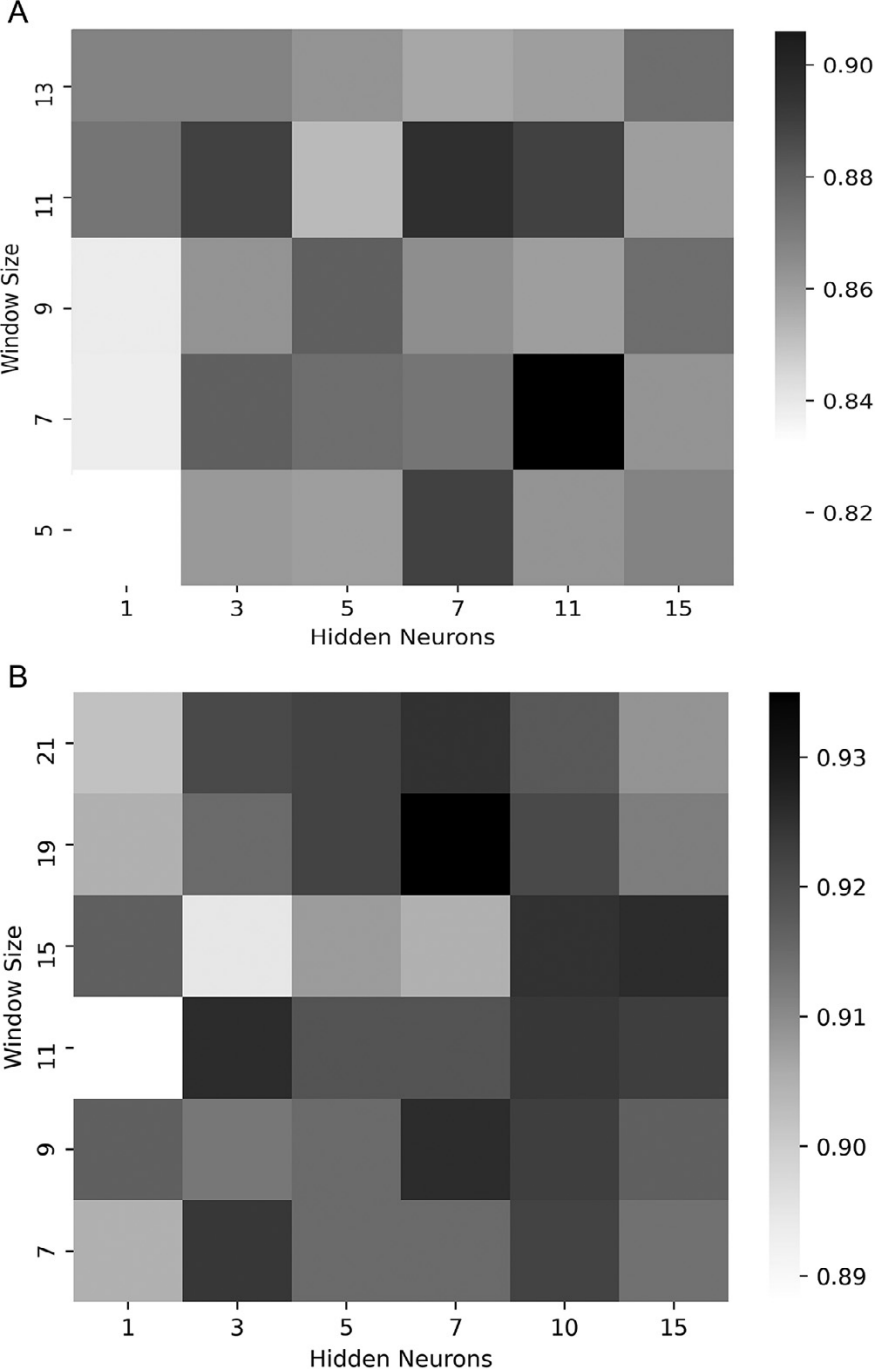
SOV as a function of window size and number of hidden neurons. A. Beta-barrel outer membrane proteins. B. Alpha-helical membrane proteins.

The choice of the optimal network size, such as the optimal number of hidden units and the window size, is key for multi-layer networks. Based on the measures of accuracy described above, the optimal parameters were identified with cross-validation. Taking into consideration the number of correctly predicted topologies, the best neural network prediction for beta barrel outer membrane proteins was obtained for neural networks that have a window size of 7 and 11 hidden neurons. Regarding alpha helical membrane proteins, beta-barrels, the optimal parameters were the window size 19 and 7 hidden neurons. These figures correlate well with the minimum length of the respective transmembrane regions (Fig. 2). Although there is little difference in accuracy for the different input contexts, the symmetric input context of one (left and right) frame seems to be slightly better than any of the other context sizes and orientations.

**Fig. 3:**
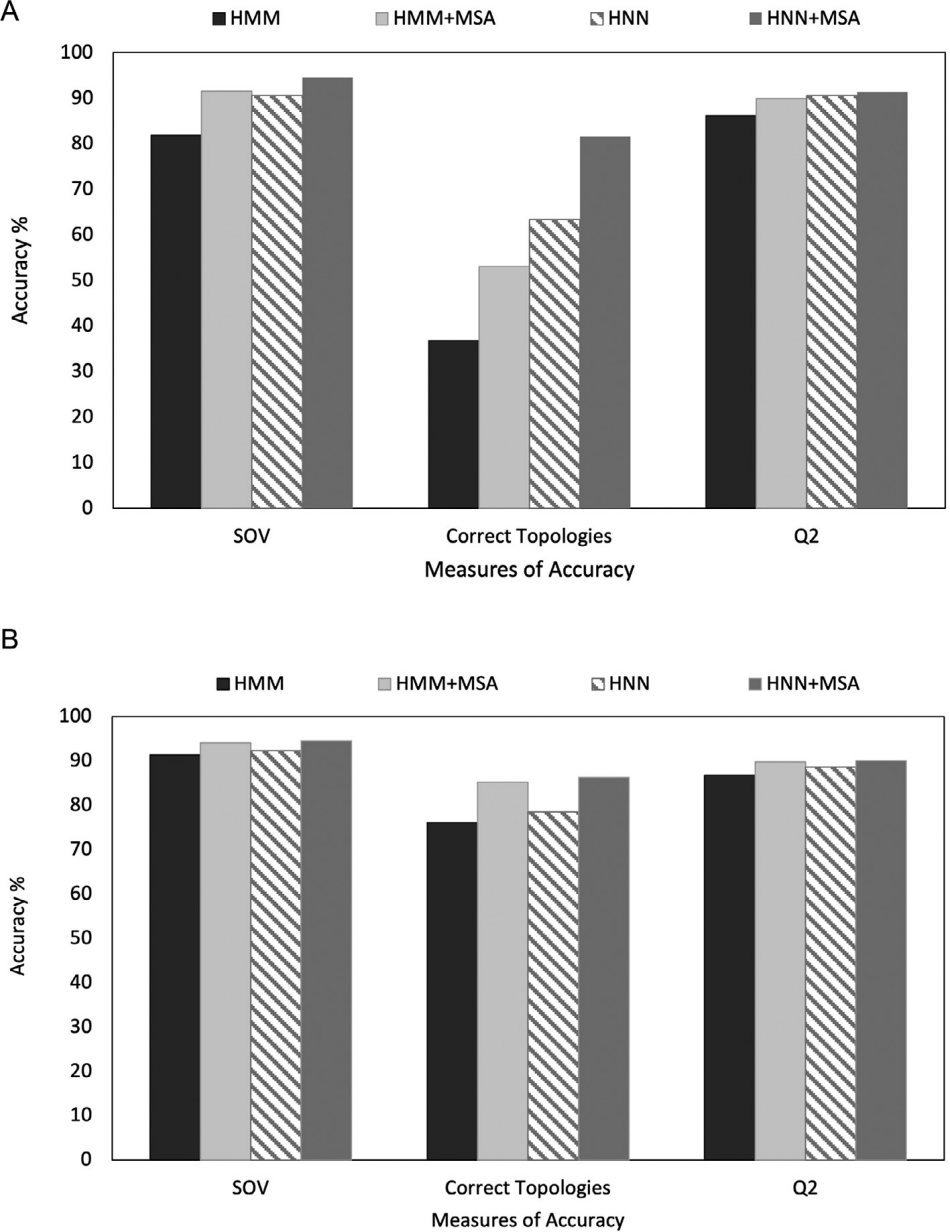
Results from the 10-fold cross validation on transmembrane protein topology prediction. A. Beta-barrel outer membrane proteins. B. Alpha-helical membrane proteins.

We present the benchmark results from the 10-fold cross-validation procedure on the transmembrane protein topology prediction in Fig. 3. It is evident that the HNN method can substantially boost the classification accuracy. More specifically, regarding beta-barrels, compared to the original PRED-TMBB2 method (2016), the increase in SOV is 8.7% and 12.7% using MSAs, the increase in correctly predicted topologies reaches 26.6% and 36.8% using MSAs and the increase in correctly predicted residues reaches 4.5% and 5.3% using MSAs. The benchmark results on the comparison against the other predictors shows that PRED-TMBB2 using HNN with the incorporation of MSAs predicts the correct topology for 40 out of 49 (81.6%) proteins (Table 1). Similar results were obtained in the blind test (Table 2). The HNN shows an improvement over HMM and the new predictor is among the top-rated ones (the reader should keep in mind that BetAware-Deep used these proteins for training). Thus, the method outperforms the majority of the currently available methods used for topology prediction of OMPs with the only possible exception the predictions for the correct topology given by BetAware-Deep, which reports a slightly higher value. We need to stress here that BetAware-Deep, is based on deep neural network architecture, and thus it is much slower regarding execution time. The per protein average execution time for the PRED-TMBB2 server was 48 s while the BetAware-Deep server took 94 s in the prediction phase, using the 49 sequences and it is not available for batch submission. Moreover, the updated method of PRED-TMBB2, shows an improved performance in discrimination where HMM slightly outperforms HNN. Using six metrics (the length of the sequence, log-odds score, log-probability, reliability score, the number of transmembrane regions, transmembrane/sequence length ratio), and applying a logistic regression classifier, the method achieves a 98.02% sensitivity on the positive set that includes 1009 OMPs derived from the OMPdb database [35], [36], and 99.06% specificity on the negative set of the 7571 non-OMPs originating from the set of Wimley. The overall MCC value is 0.96. These results compare favorably to the currently available predictors (Table 3), which include single-sequence- and the multiple-sequence-based methods, with the sole exception of HHomp, which, like BetAware-Deep, is very slow.

**Table 1:** Benchmark results on beta-barrel outer membrane proteins topology predictions.

Method	Q2	Correct Top	SOV
**PRED-TMBB2_HNN_ (this study)**	**0.914**	40	**0.946**
PRED-TMBB2 (2016)	0.880	38	0.900
BetAware-Deep	0.806	**45**	0.884
BOCTOPUS2	0.900	38	0.945
PROFtmb	0.803	29	0.832
PRED-TMBB	0.798	16	0.681

PRED-TMBB2_HNN_ results are reported based on a cross-validation test using HNN + MSA method. BetAware-Deep [32], BOCTOPUS2 [37], PROFtmb [38], PRED-TMBB [39], results were obtained using standalone versions which contain in their training set several of the proteins used in the evaluation and thus their performance is likely to be overestimated. HMM-B2TMR [40] could not complete the prediction and TMBETAPRED-RBF [41] server was not available at the time of the test.

**Table 2:** Benchmark results on beta-barrel outer membrane proteins topology predictions.

Method	Q2	Correct Top	SOV
**PRED-TMBB2_HNN_ (this study)**	0.863	**6**	0.860
PRED-TMBB2_HMM_	0.835	5	0.752
BetAware-Deep	0.903	**6**	0.879
BOCTOPUS2	0.870	**6**	**0.920**
PROFtmb	0.865	2	0.616
PRED-TMBB	0.774	1	0.598

Results are reported based on a non-redundant testing set of 7 beta barrel proteins from the 59 proteins used for training BetAware-Deep. For PRED-TMBB2HNN, PRED-TMBB2, PRED-TMBB, PROFtmb and BOCTOPUS2 these results correspond to a blind test. For BetAeare-Deep the results overestimate the performance since these proteins were included in the training set.

**Table 3:** Benchmark results on beta-barrel outer membrane proteins discrimination.

Method	MSA	Sensitivity	Specificity	MCC
**PRED-TMBB2_HNN_ (this study)**	N	98.02	99.06	**0.96**
PRED-TMBB2 (2016)	N	91.87	99.14	0.92
BetAware-Deep	N	**98.12**	97.53	0.91
BOMP	N	75.22	98.18	0.77
F-W b-Barrel Analyzer	N	97.62	90.97	0.72
PSORTb 3.0	N	59.66	98.89	0.70
TMBETADISC-RBF	N	88.90	92.22	0.69
SOSUIgramN	N	65.11	95.25	0.60
PRED-TMBB (v1)	N	69.38	92.27	0.56
TMBHunt	N	76.11	89.54	0.55
HHomp	Y	97.73	**99.95**	**0.98**
BOCTOPUS2	Y	**98.12**	98.81	0.93
PROFtmb	Y	**98.12**	84.97	0.62
BOMP-MSA	Y	78.20	98.18	0.79
SSEA-OMP	Y	96.04	88.57	0.66

PRED-TMBB2 results are reported based on a cross-validation test.

Regarding alpha-helical TM proteins, we observe an increase in SOV of 1% and 3.2% using MSAs, an increase in the correctly predicted topologies of 2.4% and 10.2% using MSAs and an increase in the fraction of correctly predicted residues of 1.8% and 3.3% using MSAs. The benchmark results on the comparison against the other predictors shows that HMM-TM using HNN with the incorporation of MSAs predicts the correct topology for 245 out of 284 (86.3%) proteins (Table 4) in a 10-fold cross-validation manner.

**Table 4:** Benchmark results on alpha-helical membrane proteins topology predictions.

Method	MSA	Q2	Correct Top	SOV
**HMM-TMv2_HNN_ (this study)**	Y	**0.901**	**245 (86.3%)**	**0.945**
HMM-TMv2 (this study)	Y	0.898	242 (85.2%)	0.940
TOPCONS	Y	0.889	236 (83.1%)	0.924
PolyPhobius	Y	0.884	219 (77.1%)	0.917
OCTOPUS	Y	0.881	220 (77.5%)	0.914
SPOCTOPUS	Y	0.881	217 (76.4%)	0.917
SCAMPI	Y	0.874	227 (79.9%)	0.911
HMM-TM (HNN)	N	0.886	223 (78.5%)	0.923
HMM-TM (HMM)	N	0.868	216 (76.1%)	0.913
TOPCONS-single	N	0.879	222 (78.2%)	0.920
TMHMM	N	0.867	197 (69.4%)	0.909
Phobius	N	0.870	194 (68.3%)	0.903
SCAMPI-single	N	0.857	164 (57.7%)	0.866
Philius	N	0.875	213 (75.0%)	0.919

HMM-TM results are reported based on a cross-validation test while TOPCONS [30], Philius [42], OCTOPUS [43], SPOCTOPUS [44], PolyPhobius [28], Phobius [45], TOPCONS-single [46], TMHMM [47], SCAMPI2 [48] results were obtained using standalone versions.

Regarding discrimination, using seven metrics (the sequence length, log-odds scores, max probability, decoder score, reliability score, number of transmembrane regions, transmembrane/sequence length ratio), and applying a logistic regression classifier, we reach a 97.54% sensitivity and 98.70% specificity (overall MCC value of 0.92) (Table 5).

**Table 5:** Benchmark results on alpha-helical membrane proteins discrimination.

Method	MSA	Sensitivity	Specificity	MCC
TOPCONS	Y	97.18	98.57	0.91
PolyPhobius	Y	98.06	95.09	0.81
OCTOPUS	Y	97.18	98.09	0.89
SPOCTOPUS	Y	**99.65**	83.83	0.55
SCAMPI	Y	97.89	97.75	0.88
**HMM-TMv2_HNN_ (this study)**	N	97.54	**98.70**	**0.92**
TOPCONS-single	N	99.65	94.60	0.78
TMHMM	N	98.84	97.51	0.90
Phobius	N	98.60	95.42	0.80
SCAMPI-single	N	95.07	97.34	0.84
Philius	N	98.94	97.37	0.87

HMM-TM results are reported based on a cross-validation test. In this test HMM and HNN had similar performance.

## 4 Discussion

Using standard HMMs, it is difficult to learn and represent long-range dependencies. The integration of a higher-order Markov chain can potentially improve the model’s performance but may come at the cost of increased computational complexity and higher number of freely estimated parameters. Therefore, it has been suggested by several authors that hybrids of HMMs and NNs may have better performance in biological sequence analysis problems. The motivation of using NNs lies in the fact that they can use as input the observation context and thereby exploit higher-order correlations between neighboring observations that can be used to improve the prediction performance. Thus, long-range dependencies can in theory better be learned and represented by HNNs than by standard HMMs. In this paper, we used the HNN approach based on Krogh’s model [8] in which all parameters are trained discriminatively at the same time by maximizing the probability of correct classification.

The Neural Network part of our HNN was a simple multilayer perceptron with one hidden layer. In future extensions, the use of more sophisticated architectures could be pursuit, such as an extension to Deep Neural Networks (DNNs). Recurrent neural networks (RNNs) and Convolutional deep neural networks (CNNs) could also be investigated, even though such approaches would require additional modifications to the HMM algorithms. Finally, the use of a pre-trained protein language model [49], [50] and fine-tune this model on the task of membrane protein topology prediction should be investigated. At least in principle, this approach should work better than HNNs because it would be able to leverage information present in the entire protein sequence, rather than a limited context *s_i_*, when predicting properties of individual amino acids.

We tested our method on the topic of topology prediction of alpha helical and beta barrel membrane proteins with encouraging results. Having chosen the strategy for initializing and training the HNN with simple emission networks, we turned towards finding the optimal architecture of these networks. We evaluated two of our previously developed methods by varying the number of hidden neurons as well as the window size. Results show significant improvement in classification when the HNN is used and the proposed method performs always better compared to a similar HMM. We also showed that the incorporation of Multiple Sequence Alignments (MSAs) can be valuable in prediction accuracy. The HNN alone has a good performance but the prediction method in terms of the topology prediction can be further improved with the incorporation of MSAs. This improvement is important as we are investigating top-scoring prediction methods, and we show that the updated versions of PRED-TMBB2 and HMM-TM outperform the currently available methods. Thus, their performance is likely to be overestimated compared to the methods presented here. Finally, we have shown that in single-sequence mode the methods developed here can be used efficiently for the identification of membrane proteins, and thus they can be valuable in order to scan entire proteomes. The updated versions of PRED-TMBB2 and HMM-TM are available at www.compgen.org. The HNN method can also be used in the context of protein sorting signals, gene-finding, prediction of functional sites in proteins and so on. We have implemented HNNs in the JUCHMME (https://github.com/pbagos/juchmme) library – an open-source CHMM library based on Java, which according to our knowledge is the only available implementation of HNNs [9].

### CRediT authorship contribution statement

**Ioannis A. Tamposis:** Software, Methodology, Validation, Investigation, Writing – original draft. **Dimitra Sarantopoulou:** Software, Methodology, Writing – review & editing. **Margarita C. Theodoropoulou:** Validation, Investigation, Writing – review & editing. **Evangelia A. Stasi:** Validation, Investigation, Writing – review & editing. **Panagiota I. Kontou:** Validation, Investigation, Writing – review & editing. **Konstantinos D. Tsirigos:** Methodology, Validation, Investigation, Data curation, Writing – review & editing. **Pantelis G. Bagos:** Software, Methodology, Validation, Data curation, Writing – review & editing, Conceptualization, Supervision, Funding acquisition.

## Declaration of Competing Interest

The authors declare that they have no known competing financial interests or personal relationships that could have appeared to influence the work reported in this paper.
